# Tobacco price elasticity by socioeconomic characteristics in Ecuador

**DOI:** 10.1371/journal.pone.0302293

**Published:** 2024-04-19

**Authors:** Ana Cristina Mena, Guillermo Paraje

**Affiliations:** 1 Business School, Universidad de Las Américas, Escuela de Negocios, Quito, Ecuador; 2 Business School, Universidad Adolfo Ibanez, Santiago de Chile, Chile; Universidad San Francisco de Quito, ECUADOR

## Abstract

Smoking is a worldwide epidemic and increased prices are one of the most cost-effective measures to reduce tobacco consumption. This article aims to estimate the price and income elasticity of cigarettes for different population groups in Ecuador. The National Survey of Urban and Rural Household Income and Expenditures (ENIGHUR) 2011–2012 was used, which has information on household cigarette consumption and its sociodemographic characteristics. Deaton’s Almost Ideal Demand System, which decouples the effect of quality on the price of the good, was applied. The elasticities were calculated for several groups: urban/rural, income levels (tertiles), education level, sex and age ranges of the household head, and frequency of cigarette purchases in households. The estimated price elasticity nationwide is -0.89 and the income elasticity is 0.41, both statistically significant. Households headed by women (-2.22) are more sensitive to an increase in cigarette prices than those headed by men (-0.65) and households headed by people between 20 and 40 years of age (-2.32) have a higher price elasticity compared to country-level estimations. Differences within other groups are not statistically significant.

## Introduction

Smoking is defined as a worldwide epidemic [1]. It is the leading cause of preventable death [2]. More than six million people die each year worldwide due to tobacco use [3]. According to the World Health Organization, in 2015 there were around 1.1 billion people who smoke, representing 20% of the population over 15 years of age worldwide [1]. In Latin America and the Caribbean there are 69.10 million people who smoke, with tobacco accounting for 9.40% of deaths in the region [3]. Although tobacco use has decreased since 1980 by about 10 percentage points, deaths related to this risk factor have increased since 1990 in the region. In Ecuador, it is estimated that 13.40% of all deaths registered in the country in 2015 can be attributed to tobacco, i.e., 7,798 preventable deaths [4].

In Ecuador, in 2018, the prevalence of current tobacco use among adults was 13.70%. In line with international trends, cigarette consumption in Ecuador is higher in men than in women. The prevalence of current tobacco use was 23.80% among males and 4.00% among females [5]. Current consumption in adults in 2012 was higher in rural areas, in the Afro-Ecuadorian population and in the poorest quintile [6]. In the population aged 10 to 19 years, the prevalence of current tobacco use was 28.40% in 2012.

The prevalence of daily tobacco use among adults was 3.50% in 2021, with notable differences between men (6.30%) and women (0.90%) [5].

As part of compliance with the Framework Convention on Tobacco Control, in 2007, the implementation of aggregated measures such as MPOWER (Monitor, Protect, Offer, Warn, Enforce and Raise) was initiated. They are an instrument to aid countries in implementing actions to reduce tobacco consumption. One of these measures is the raising of tobacco taxes [5, 7].

There is evidence showing that taxation is the most effective measure to reduce tobacco consumption [8–10]. According to estimates, a more than four-fold increase in the price of tobacco worldwide would have reduced the prevalence of tobacco use to 15,0% in 2015, thus hastening the population health outcomes [3]. Ecuador has been recognized internationally for its tobacco taxation policy [11] that has made cigarettes less affordable since 2012 and represent a relevant proportion in cigarettes retail price, close to World Health Organization recommendations [5].

Studies of tobacco demand show that there is a negative, inelastic price elasticity, i.e. with a less than proportional reduction in consumption in response to price increases. In a systematic review of studies conducted in Latin America and the Caribbean, Guindon et al. estimated elasticities between -0.31 and -0.43 in the short and long term [12] in developed countries, while in developing countries it may be between -0.40 and -0.80 [13].

The response of the population to a price increase can be variable depending on several factors. John estimated the price elasticities of different tobacco products in the range of -0.34 to -0.91 [14], finding a more elastic demand for the consumption of *bidis* and tobacco leaves. Selvaraj et al. estimated price elasticities for various tobacco products among economic classes in India. They found higher price sensitivity in poorer households for cigarettes (-0.83), bidis (-0.43) and leaf tobacco (-0.56) indicating that poor people are more responsive to price changes. In contrast, the richest people have the lowest price elasticity for bidis (-0.08) and tobacco leaf (-0.05) compared to the first and second tertiles [15].

For Ecuador, Chávez estimates the price elasticity of cigarette consumption in general and also differentiated by income level and sex of the household head [16]. The results suggest a higher elasticity in households with a higher level of consumption (income proxy). These estimates use the methodology developed by Deaton [17, 18] but limit the definition of clusters to the number of parishes and do not consider other relevant population disaggregation that could contribute to the definition of public policies prioritizing populations with higher risk levels.

Given the differences in consumer preferences, the analysis of the price effect on tobacco demand can be performed by differentiating household education levels, income or available assets, place of residence, among others [19]. This separation seeks to determine the variable responses of the population to tobacco tax measures and their potential outcomes.

The objective of this article is to estimate the price and income elasticity of cigarettes in Ecuador considering different population groups according to their sociodemographic and tobacco consumption characteristics. Estimates available for Ecuador consider only income level and sex of household head.

## Materials and methods

### Data

The National Survey of Urban and Rural Household Income and Expenditures 2011–2012 (ENIGHUR) from the National Institute of Statistics and Census (INEC) was used. This is the latest available national survey on household budgets. The survey includes information from 39,617 households nationwide, in urban and rural areas. The sample was drawn taking into account the 2010 census cartographic update. The study domains correspond to the 24 urban and rural provinces and 9 self-represented cities. In the cities, the sample selection was probabilistic and two-stage, with the census sectors being the primary sampling units (PSU) and the dwellings the secondary units [20].

For the application of the demand system, the census sectors were considered as clusters. The households surveyed for ENIGHUR were distributed in 3411 census sectors. This survey provides detailed information on total household expenses, including specific products like tobacco. It disaggregates data by quantities and monetary values, and collects sociodemographic data at both individual and household levels. These characteristics make ENIGHUR particularly strong for applying the selected method to estimate elasticities, as described in further detail.

ENIGHUR provides information on monthly household money expenditure on cigarettes, quantity consumed (units) and total household current expenditure. The figures correspond to the period in which the information was collected, with mensualized and standardized data for household expenditures [20]. The quantity of cigarettes purchased was mensualized based on the reported frequency of purchase.

The survey made it possible to extract sociodemographic variables on household structure (number of members, participation of women, etc.), and on the characteristics of the head of household (age, sex, level of education, ethnicity, etc.), as well as household spending on a variety of items. Finally, the variable household cigarette purchase frequency was defined, which refers to cigarette consumption in households.

ENIGHUR shows that household spending on cigarettes is higher in urban areas when the household head is male, has higher education or is Afro-Ecuadorian as shown in Table 1.

**Table 1:** Sociodemographic structure, average cigarette expenditure, average total household expenditure and unit price of cigarettes by population groups.

	Socio-demographic structure (%)	Unit price per cigarette (US dollar)	Share of cigarette spending in total household spending	Total household spending (annual, US dollar)	Household spending on cigarettes (monthly, US dollar)	Number of cigarettes(monthly)
Rural Area	32.02	0.156	0.028	6 313.36	8.97	73
Urban Area	67.98	0.159	0.016	11 317.47	10.99	94
*Sex of household head*						
Female	23.85	0.162	0.016	8 407.29	9.85	74
Male	76.15	0.157	0.020	10 125.95	10.54	91
*Education level of the household head*						
No education	8.10	0.152	0.035	5 018.51	10.58	81
Elementary	44.38	0.155	0.023	7 272.02	9.42	78
High School	29.89	0.162	0.017	10 034.63	10.07	89
University	17.62	0.159	0.013	17 488.96	12.95	110
*Ethnicity of household head*						
Mestizo	78.02	0.160	0.017	10 227.93	9.99	84
Indigenous	6.84	0.175	0.015	5 855.56	6.14	41
Afro-Ecuadorian	5.05	0.143	0.035	8 264.23	12.64	108
Other	10.09	0.148	0.032	9 093.32	14.10	130
*Country-wide*	* *	0.158	0.020	9 715.36	10.44	89

^a^ Total household expenditure was estimated for the national total. Source: ENIGHUR 2011–2012.

### Method

Elasticities are estimated by applying the Almost Ideal Demand System (AIDS) proposed by Deaton and Muellbauer (1980) [17] and the adjusted model proposed by Deaton (1988) [18].

Deaton’s method [17, 18] allows estimating the elasticities of demand by decoupling the effect of the quality of goods on price. It assumes variation in prices between geographic areas, due to transportation costs, border prices or others, variation that serves as an instrument to correct endogeneity. In the absence of information on prices at the geographic level, unit values are used as a proxy for price [18], calculated for each household, by relating spending on cigarettes to the purchased amounts. With the ANOVA analysis, a significant F value of 1.7 and an R^2^ of 0.6 were obtained, so that the unit values can be used for the proposed analysis, since they vary between clusters (census sectors) but not within each cluster [19].

The equation system contains a demand function (cigarette consumption share) and a unit value function for each cluster, as follows:

$$w_{hc}=\propto_{0}+\beta_{0}{lng}_{hc}+\gamma_{0}X_{hc}+\omega_{0}{ln\pi }_{c}+(f_{c}+\mu_{0hc})$$ (1)

$${lnp}_{hc}=\propto_{1}+\beta_{1}{lng}_{hc}+\gamma_{1}X_{hc}+\omega_{1}{ln\pi }_{c}+\mu_{1hc}$$ (2)

Where *w* is the share of household expenditure *h* in the cluster *c*, *lng* is the logarithm of total household expenditure, *X*_*hc*_ is a vector of socio-demographic characteristics of households and of the household head, *μ*_*hc*_ is the error term that is added to the cluster fixed effects represented by *f*_*c*_. Eq (2) corresponds to the price equation, where *lnp*_*hc*_ is the natural logarithm of the unit value of each household in each cluster and the other variables are kept as in Eq (1). The *f*_*c*_ term is not included in the unit price regression because the unit values do not depend on cluster fixed effects, but on the quality of the goods. Variable *lnπ*_*c*_ are the unobserved prices, which are not included in the estimations of the equations but are estimated in the subsequent stages of the model [17–19].

Unit value is calculated as the quotient of buyers’ monthly expenditure on cigarettes and the monthly purchased quantity. Total household expenditure corresponds to total purchases plus annualized observed imputed household expenditure. The sociodemographic variables included in the equations are the natural logarithm of the number of household members, ratio of women in the household, ratio of children under 15 years of age in the household, sex and age of the household head, dummy variable of ethnic self-identification of the household head (indigenous, Afro-Ecuadorian and mestizo) and dummy variable of the level of completed education of the household head. The estimations are made for households with positive cigarette consumption.

Price and expenditure elasticities are estimated for the population in urban and rural areas, by income tercile, ranks by age, sex, and education level of the household head, and by frequency of cigarette purchases by household. For the classification of households by income, we used the classification proposed by Paraje [21] using income tertiles. Households were grouped according to the age of the household head into under 20 years old, 21 to 40 years old, 41 to 65 years old and over 65 years old. The frequency of cigarette purchases by households was grouped into three ranges: daily and weekly frequency, biweekly and monthly frequency, and frequency greater than monthly (quarterly, semiannual and annual).

The above-described system of equations was estimated for each population group. In each case, ANOVA analysis was performed to verify price variation between and within clusters. In all cases included in the analysis, a significant F and an R^2^ greater than 0.5 were obtained, with the exception of the group with biweekly and monthly purchase frequency and with a household head of household under 20 years old. In this way, the parameters suggested for the use of unit values in the estimation of elasticities are complied with [19].

Using the obtained results, the *z-score* of the differences in each sociodemographic characteristic was calculated to test the statistical difference between groups in cases where confidence intervals did not overlap. The formula applied is the following:

$$z-score=\frac{\beta_{1}{-\beta }_{2}}{\sqrt{\varepsilon_{1}^{2}{+\varepsilon }_{2}^{2}}}$$ (3)

Where *β*_1_ and *β*_2_ are the coefficients of the variables in each of the equations applied per sociodemographic characteristic, and *ε*_1_ and *ε*_2_ are the respective standard errors. This indicator evaluates the difference between the different population groups. For the comparison of the elasticities, we calculated the urban and rural value, first and third terciles, first and second tertile, household head from 20 to 40 years old and over 65 years old, male and female household head, and higher and primary education of the household head. Z-scores were calculated for groups where confidence intervals of their categories did not overlap. The obtained Z-scores were compared to the critical value of 1.96 for a 95% confidence level; if a greater absolute value was obtained, the null hypothesis (*β*_1_ = *β*_2_) was rejected, indicating that the elasticities were different.

Data and demand system processing was performed in Stata 18.0, and standard errors were estimated using Bootstrap after 1,000 replications.

## Results

Table 2 shows the results of the income elasticity and own price elasticity of cigarettes at the country level and for the selected population groups, using the demand system proposed by Deaton. The standard error, the significance level of the obtained results and the confidence interval are also presented (95%).

**Table 2:** Conditional cigarette price-elasticity and expenditure elasticity by socioeconomic group.

	Elasticity	Standard error		Confidence interval (95%)		Data
				lb	ub	
**Total**						
Expenditure elasticity	0.412	0.088	(***)	0.239	0.585	5063
Price elasticity	-0.899	0.177	(***)	-1.245	-0.552	3410
**Urban area**						
Expenditure elasticity	0.561	0.096	(***)	0.372	0.750	3999
Price elasticity	-0.714	0.197	(***)	-1.101	-0.328	3410
**Rural area**						
Expenditure elasticity	-0.215	0.201		-0.609	0.179	1064
Price elasticity	-0.877	0.188	(***)	-1.246	-0.509	3410
**First income tertile**						
Expenditure elasticity	-1.328	0.368	(***)	-2.050	-0.606	923
Price elasticity	-1.592	0.792	(**)	-3.144	-0.040	3410
**Second income tertile**						
Expenditure elasticity	1.073	0.324	(***)	0.438	1.708	1599
Price elasticity	-0.536	3.701		-7.789	6.717	3410
**Third income tertile**						
Expenditure elasticity	0.717	0.128	(***)	0.466	0.968	2541
Price elasticity	-1.343	0.303	(***)	-1.937	-0.748	3410
**Head of household age: 20 to 40 years old**						
Expenditure elasticity	0.857	0.201	(***)	0.463	1.251	1714
Price elasticity	-2.322	0.591	(***)	-3.481	-1.162	3410
**Head of household age: 40 to 65 years old**						
Expenditure elasticity	0.285	0.195		-0.097	0.666	2689
Price elasticity	-1.543	0.443	(***)	-2.412	-0.675	3410
**Head of household age: over 65 years old**						
Expenditure elasticity	-0.768	0.684		-2.110	0.573	617
Price elasticity	-1.272	0.154	(***)	-1.573	-0.970	3410
**Sex of household head: male**						
Expenditure elasticity	0.330	0.104	(**)	0.126	0.534	4343
Price elasticity	-0.654	0.231	(***)	-1.107	-0.200	3410
**Sex of household head: female**						
Expenditure elasticity	0.730	0.618		-0.482	1.942	720
Price elasticity	-2.229	0.331	(***)	-2.878	-1.581	3410
**Education level of household head: elementary school**						
Expenditure elasticity	-0.021	0216		-0.444	0.401	2065
Price elasticity	-0.984	0.257	(***)	-1.487	-0.482	3410
**Education level of household head: high school**						
Expenditure elasticity	0.657	0.252	(**)	0.164	1.150	1606
Price elasticity	0.001	0.483		-0.946	0.948	3410
**Education level of household head: university**						
Expenditure elasticity	0.601	0.234	(*)	0.143	1.060	1123
Price elasticity	2.921	33.425		-62.590	68.432	3410
**Frequency of household cigarette purchase: daily and weekly**						
Expenditure elasticity	0.331	0.092	(***)	0.150	0.512	4482
Price elasticity	-0.792	0.105	(***)	-0.997	-0.586	3410
**Frequency of household cigarette purchase: biweekly and monthly**						
Expenditure elasticity	0.787	0.967		-1.108	2.682	529
Price elasticity	-1.804	38.235		-76.744	73.136	3410

(***) Significative at 99% (**) Significative at 95% (*) Significative at 90%

^a^ Standard errors are obtained by Bootstrap after 1000 replications

Source: ENIGHUR 2011–2012.

The estimated elasticities for households with a household head under 20 years of age and with a purchase frequency greater than monthly recorded few observations for the estimates, with no results obtained. The own price elasticities estimated are negative and significant at the 99% confidence level for both urban and rural areas, the third income quintile, household heads of various ages, male and female household heads, household heads with primary education, and households making daily and weekly cigarette purchases. The own price elasticity for the first income tertile is significant at the 95% confidence level.

Regarding income elasticity, almost all socioeconomic groups have a positive sign and significant elasticities, except for the first income tertile group, which shows a negative income elasticity. Additionally, in rural areas, households headed by individuals aged 20 to 40 and over 65 years old, households with female heads, households with elementary education, and households making biweekly and monthly cigarette purchases, the elasticities are not significant.

At the country level, the estimated price elasticity is -0.89, which corresponds to an inelastic demand (i.e., a 10% increase in prices reduces demand for cigarettes in 8.9%) These results show that an increase in cigarette prices generates a decrease in demand proportionally smaller than the percentage change in prices. The income elasticity is 0.41 (i.e., a 10% increase in income increases demand for cigarettes in 4.1%). This result indicates that, with greater availability of resources, households tend to increase spending on cigarettes, but to a lesser extent than the income increase, indicating that cigarettes are normal goods.

The comparison of confidence intervals between the different categories of a group (e.g., rural and urban for area of residency) revealed significant differences in almost all cases for expenditure elasticity, highlighting diverse spending behaviors in response to income changes. However, confidence intervals for gender groups overlapped, as did measures for subgroups based on the education level of the household head. Only for gender groups did the absolute value of the z-score fall below the critical threshold of 1.96 at a 95% confidence level. This suggests that, unlike other demographic categories, there was no statistically significant difference in expenditure elasticity between genders.

A distinct pattern emerged concerning price elasticity, with statistically significant differences found only within the gender category. In this case, confidence intervals did not overlap, indicating a statistically significant difference in price sensitivity between men and women. Specifically, women exhibited a higher sensitivity to price fluctuations compared to men, suggesting that price changes have a more pronounced impact on tobacco purchasing decisions for women. The results indicate that for the rest of groups there are no significant differences in price elasticities within the categories of each group because their confidence intervals did overlap and the absolute values of the z-scores estimated were below the critical value (Table 3). However, households with a head aged between 20 and 40 exhibit a price elasticity higher than the national average, with a statistically significant difference. This suggest that households with younger heads are more sensitive to increases in cigarette prices compared to the country-level estimations.

**Table 3:** Z-scores for elasticity differences.

	Z-Scores	
	Expenditure elasticity	Price elasticity
Urban/Rural	-	0,60
First/third tertile	-	-0,29
First/second tertile	-	-0,28
Household head 20–40 years old / 40–65	-	-1,72
Male / female household head	-0,64	-
Higher / elementary education household head	-1,96	-0,12

Source: ENIGHUR 2011–2012.

## Discussion

The results of the elasticities obtained for Ecuador, based on a robust and widely used methodology for estimating demand for tobacco and other products, show an inelastic demand in the face of price variations, although close to unity. The relationship between prices and cigarette consumption is consistent with that found in other countries of the region [13], although to a greater extent.

The price elasticity estimated in this study is similar to that obtained in another study conducted for Ecuador based on household surveys, where an elasticity of -0.87 was obtained [16]. However, the income elasticity is lower than that estimated in the previous study. Additional differences are found in the estimated price elasticities for the sex of the household head and socioeconomic level.

These differences may be due to the methodological parameters used for the estimates. Chavez [16] defined the clusters based on the territorial division into 624 parishes, the socioeconomic level was stratified based on expenditure, and the average unit values show differences with the present study, which would explain the changes in the estimates obtained. In this study, the number of clusters is extended to allow for more consistent estimates of the parameters [19] and to ensure that households within the cluster are in the same market and have little variation in unit prices [19, 22].

Other estimates for Ecuador with time series calculate negative price elasticities between -1.96 [23] and -0.46 [24]. Some methodological limitations of using time series to estimate demand behavior are acknowledged. The use of series with short time periods, monthly or quarterly, may affect the estimates, as well as the limited use of control variables or the bias that may be generated by the existence of tax evasion when using legal sales information [12]. Other limitations of this type of estimation arise because the information used does not allow for data at the individual level, restricting the evidence even more for elasticities given by socioeconomic characteristics, such as age, sex and income level [12].

This study provides estimates of elasticities for other demographic characteristics such as education level and age of household head, consumption level based on frequency of tobacco purchase and area of residence, since such estimates have not been previously made for Ecuador. The results obtained would indicate differences in elasticity between the groups, although the results should be interpreted with caution given the limitations of the methodology for comparing subgroups. This generates evidence on the effects that cigarette tax increases may have on different population groups, taking into account how much of the price increase is passed on to the consumer. The estimated differences are of utmost importance for the definition and effectiveness of public policies aimed at tobacco control, particularly measures that lead to an increase in tobacco prices.

The results indicate significant differences in the households’ response to price changes depending the gender of the household head. Price elasticity es higher when a household is headed by a woman. This result may be attributed to differences in consumption prevalence between men and women. Evidence regarding price elasticity by gender is limited. Some studies conclude that women are more sensitive to price changes than men, particularly among younger age groups [25–27]. However, other studies suggest that men have a higher price elasticity in tobacco consumption [28, 29].

The higher responsiveness of households with relatively young heads provides important insights into the effects of cigarette price interventions on consumption among the young population. These results are consistent with other studies [30] that show that tax increases are an effective tool for reducing consumption among youth and young adults.

The study did not find statistically significant differences between households with different income levels. Evidence for other countries shows that households with lower incomes tend to exhibit higher price elasticities [30]. In India, for instance, the poorest households are the most price sensitive [15]. Chavez’s study [16] estimates a higher elasticity for households with higher incomes in Ecuador; however, differences between groups were not tested. In Colombia, Gallego *et al*. find almost no differences across socioeconomic level [27]. In high-income countries, there is evidence that indicate that groups with lower income or education are more sensitive to price changes [31]; nevertheless, evidence for low-and-middle-income countries is too limited and weak to draw conclusions about differences between groups [32].

The results address the response of individuals on cigarette consumption to a price increase. This price increase is related to the tax increase. However, the effect of a tax measure on consumption depends on how the increase is passed on to consumers. Consumer response may also be affected by changes in market structure due to substitution by lower-cost brands or by the growth of illicit trade.

Limited information from household surveys that reveal detailed information on household income and expenditures in turn limits the analysis of the most recent changes in demand behavior. Estimates are for the period 2011–2012, as these are the latest available data on household budgets. This period also coincides with important changes to the national cigarette tax. In 2011, the cigarette tax was changed from an ad valorem rate to a specific rate per unit, which has been steadily increased until today [19]. Moreover, changes in country income and growth in last decade could have influenced consumers’ responsiveness to price changes [33]. This raises the need for more current data to detect changes in consumer behavior, as well as potential differentiated responses to possible changes in market structure, such as the presence of illicit trade. Future research efforts could be oriented towards analyzing demand behavior at different price levels and how consumer response to market changes has evolved.

A deeper understanding of the population’s response to cigarette smoking can contribute to the definition of health policies that take into account the specificities of the population on which they affect. Consumer preferences and the characteristics of their home and environment contribute to differentiated responses, and therefore differences in the obtained results.
